# Simple Method of Arresting Hemorrhage from Leech-Bites

**Published:** 1845-06

**Authors:** Lewis Hensley


*Simple Method of Arresting Hæmorrhage from Leech-Bites.*—In the Lancet of February 1st, Mr. Hogg records a case, wherein he experienced much difficulty in stopping the bleeding of leech-bites in a child, and was obliged to have recourse to the actual cautery. As this is a difficulty of not unfrequent occurrence, it may be useful to mention that I have succeeded in arresting active bleeding from a leech-bite, by encircling the bite with a section of quill, about the eighth of an inch in height, and fixed in its situation by adhesive plaster. I place a fold of lint over the orifice, to prevent the edge of the quill being too sharp, and the little circle, so as to include the bite in its centre, over that, a compress and adhesive plaster.—*Lewis Hensley.*

